# Supplementary material for: Enhanced Cardiorenal Protective Effects of Combining SGLT2 Inhibition, Endothelin Receptor Antagonism and RAS Blockade in Type 2 Diabetic Mice
Source: Int J Mol Sci. 2022 Oct 24;23(21):12823. doi: 10.3390/ijms232112823 (PMC9656616; doi:10.3390/ijms232112823)
Supplement: Supplementary file 1 [file ijms-23-12823-s001.zip › ijms-1935255-supplementary.pdf]

Supplemental Material

Enhanced cardiorenal protective effects of combining SGLT2 inhibition, endothelin receptor antagonism and RAS blockade in type 2 diabetic mice

by

Ander Vergara[1,2,*], Conxita Jacobs-Cacha[1,*], Carmen Llorens-Cebria[1], Alberto Ortiz[3], Irene Martinez-Diaz[1], Nerea Martos[1], Pamela Dominguez Báez[1], Mireia Molina Van den Bosch[1], Sheila Bermejo[1,2], Michael Paul Pieper[4], Begoña Benito[5,6,7,†], and Maria Jose Soler [1,2,†].

[1]Nephrology and Kidney Transplantation Research Group. Vall d'Hebron Institut de Recerca (VHIR). Passeig Vall d'Hebron 119-129, 08035, Barcelona, Spain.
[2]Nephrology Department. Vall d'Hebron Hospital Universitari. Passeig Vall d'Hebron 119-129, 08035, Barcelona, Spain.
[3]IIS-Fundación Jiménez Diaz, Universidad Autónoma de Madrid, Fundación Renal Iñigo Álvarez de Toledo-IRSIN, REDinREN, Instituto de Investigación Carlos III. Av. de los Reyes Católicos 2, 28040, Madrid, Spain.
[4]Cardio-metabolic Diseases Research. Boehringer Ingelheim Pharma GmbH & Co KG. Birkendorfer Str. 65, 88397, Biberach an der Riß, Germany.
[5]Cardiology Research Group. Vall d’Hebron Institut de Recerca (VHIR), Vall d'Hebron Hospital Universitari, Vall d’Hebron Barcelona Hospital Campus. Passeig Vall d'Hebron 119-129, 08035, Barcelona, Spain.
[6]Cardiology Department. Vall d'Hebron Hospital Universitari. Passeig Vall d'Hebron 119-129, 08035, Barcelona, Spain.
[7]Deparment of Medicine. Universitat Autònoma de Barcelona. Av. de Can Domènech, 08193, Bellaterra, Spain.

* These authors contributed equally to the work and share the first authorship.
† Senior and corresponding authors.

TABLE OF CONTENTS

TABLES 4

Table S1. Additional echocardiographic parameters of vehicle-treated db/m mice, vehicle-treated db/db mice and db/db mice treated with empagliflozin, atrasentan, ramipril or their combinations. 4

Table S2. Primer sequences used for SYBR Green real-time quantitative PCR (RT-qPCR). 5

Table S3. Number of mice included and Tukey's outlier values in each of the measurements performed during the study. 6

FIGURES 9

Figure S1. Blood pressure before and after treatment in vehicle-treated db/m mice, vehicle-treated db/db mice and db/db mice treated with empagliflozin, atrasentan, ramipril or their combinations. 9

Figure S2. Glomerular filtration rate before and after treatment in vehicle-treated db/m mice, vehicle-treated db/db mice and db/db mice treated with empagliflozin, atrasentan, ramipril or their combinations. 10

Figure S3. Water intake after treatment in vehicle-treated db/m mice, vehicle-treated db/db mice and db/db mice treated with empagliflozin, atrasentan, ramipril or their combinations. 11

Figure S4. Renal tubulointerstitial fibrosis measured by picrosirius red staining in vehicle-treated db/m mice, vehicle-treated db/db mice and db/db mice treated with empagliflozin, atrasentan, ramipril or their combinations 12

Figure S5. Evaluation of renal mRNA expression and kidney and serum activity of angiotensin-converting enzyme (ACE) and angiotensin-converting enzyme 2 (ACE2) in vehicle-treated db/m mice, vehicle-treated db/db mice and db/db mice treated with empagliflozin, atrasentan, ramipril or their combinations 13

Figure S6. Experimental design. 14

Figure S7. Mice death or excluded during the experimental procedure. 15

TABLES

Table S1. Additional echocardiographic parameters of vehicle-treated db/m mice, vehicle-treated db/db mice and db/db mice treated with empagliflozin, atrasentan, ramipril or their combinations.

	db/m	db/db	db/db EMP	db/db EMP+RAM	db/db EMP+RAM+ATR	db/db ATR	db/db ATR+RAM	db/db RAM
**Aortic diameter (mm)**	1.50 (1.44-1.55)	1.52 (1.38-1.68)	1.48 (1.44-1.66)	1.40* (1.35-1.42)	1.35* (1.34-1.46)	1.38 (1.35-1.50)	1.54 (1.42-1.61)	1.48 (1.43-1.51)
**LVPW thickness (mm)**	0.97 (0.91-0.98)	0.99 (0.87-1.10)	0.87* (0.85-0.96)	0.89* (0.84-0.93)	1.03 (0.87-1.06)	0.91 (0.80-0.95)	0.89* (0.77-0.92)	0.96 (0.92-1-03)
**Fractional shortening (%)**	35.5 (33.8-38.7)	36.3 (35.0-37.0)	38.0 (34.3-40.0)	37.9 (35.6-42.0)	38.6 (35.0-40.8)	34.7 (32.8-36.0)	39.9* (35.6-40.9)	36.7 (36.3-39.0)
**E/A ratio**	2.40 (2.29-2.52)	1.99$ (1.92-2.14)	2.08 (1.38-3.07)	0.93* (0.48-1.63)	1.44 (1.00-1.93)	1.99 (1.84-2.19)	0.86* (0.60-1.68)	1.51* (0.61-2.08)

LVPW: left ventricle posterior wall. **db/m**: non-diabetic mice treated with vehicle. **db/db**: diabetic mice treated with vehicle. **db/db EMP**: diabetic mice treated with empagliflozin. **db/db EMP+RAM**: diabetic mice treated with empagliflozin and ramipril. **db/db EMP+RAM+ATR**: diabetic mice treated with empagliflozin, ramipril and atrasentan. **db/db ATR**: diabetic mice treated with atrasentan. **db/db ATR+RAM**: diabetic mice treated with atrasentan and ramipril. **db/db RAM**: diabetic mice treated with ramipril. Values are shown as median and interquartile range (Q1-Q3). $ $p<0.05$ vehicle-treated db/db mice vs vehicle-treated db/m mice. * $p<0.05$ any treated db/db mice compared with vehicle-treated db/db mice.

Table S2. Primer sequences used for SYBR Green real-time quantitative PCR (RT-qPCR). The present table displays protein and gene names for each of the genes analyzed, the forward and reverse primer sequence, as well as the dilution employed in each case.

Protein	Gene	Forward primer (5′-3′)	Reverse primer (5′-3′)	cDNA dilution
Angiotensin converting enzyme	Ace	CGCCGCTATGGGGACAAATA	ATGTCTCCCAGCAAATGGGC	1/25
Angiotensin converting enzyme 2	Ace2	CGCAGAGATCAAGCCATTGT	TCCATCAACTTCCTCCTCACA	1/25
Angiotensinogen	Agt	CGTGCCCCTAGGTGAGAGAG	TCCAAGTCAGGAGGTCGTTC	1/25
Collagen I alpha-1 chain	Col1a1	GAGCGGAGAGTACTGGATCG	GTTCGGGCTGATGTACCAGT	1/50
Hypoxanthine-guanine phosphoribosyltransferase	Hprt1	TGTTGTTGGATATGCCCTTG	AATGACACAAACGTGATTCAAA	1/25
α-Myosin heavy chain	Myh6	CCAACACCAACCTGTCCAAGT	AGAGGTTATTCCTCGTCGTGCAT	1/50
β-Myosin heavy chain	Myh7	CTCAAGCTGCTCAGCAATCTATTT	GGAGCGCAAGTTTGTCATAAGT	1/50
NADPH oxidase 4	Nox4	CTTGGTGAATGCCCTCAACT	TTCTGGGATCCTCATTCTGG	1/100
Renin	Ren1	ACCTTGCTTGTGGGATTCAC	CCTGATCCGTAGTGGATGGT	1/25

Table S3. Number of mice included and Tukey's outlier values in each of the measurements performed during the study.

	db/m	db/db	db/db EMP	db/db EMP+RAM	db/db EMP+RAM+ATR	db/db ATR	db/db ATR+RAM	db/db RAM
**Body weight**								
Animals included	12	15	14	15	15	8	13	9
Outliers	-	-	-	-	-	-	-	1
**Blood glucose**								
Animals included	12	15	14	15	15	8	13	9
Outliers	-	1	4	-	-	1	2	1
**Body fat**								
Animals included	12	15	14	15	15	8	13	9
Outliers	-	-	-	-	-	-	1	-
**Subcutaneous fat**								
Animals included	12	15	14	15	15	8	13	9
Outliers	-	1	-	-	-	-	1	-
**Intraabdominal fat**								
Animals included	12	15	14	15	15	8	13	9
Outliers	-	-	-	-	-	-	-	-
**Blood glucose**								
Animals included	12	15	14	15	15	8	13	9
Outliers	-	1	4	-	-	1	2	1
**Systolic BP pre**								
Animals included	12	15	14	15	14	8	13	9
Outliers	-	-	-	-	-	-	1	-
**Diastolic BP pre**								
Animals included	12	15	14	15	14	8	13	9
Outliers	-	-	1	-	-	-	-	-
**Systolic BP post**								
Animals included	12	15	14	15	14	8	10	9
Outliers	-	-	-	-	1	-	-	-
**Diastolic BP post**								
Animals included	12	15	14	15	14	8	10	9
Outliers	-	-	-	-	-	-	-	-
**Heart rate post**								
Animals included	12	15	14	15	14	8	10	9
Outliers	-	2	-	-	-	-	-	-
**GFR pre**								
Animals included	12	15	14	14	15	8	13	9
Outliers	-	-	-	1	-	-	-	-
**GFR post**								
Animals included	9	12	8	9	9	6	8	7
Outliers	2	-	-	-	-	-	-	-
**GFR difference**								
Animals included	9	12	8	9	9	6	8	7
Outliers	-	1	1	-	-	1	-	-
**UACR**								
Animals included	11	14	14	15	15	8	13	9
Outliers	1	1	2	-	-	1	1	-
**Urine/Plasma glucose**								
Animals included	10	10	11	11	11	8	10	8
Outliers	1	-	-	-	1	-	-	2
**Water intake**								
Animals included	12	15	14	15	15	8	13	9
Outliers	1	1	-	-	1	-	-	1
**Mesangial matrix expansion**								
Animals included	11	11	11	11	11	8	9	8
Outliers	-	2	-	-	-	-	-	-
**Podocyte density**								
Animals included	10	11	10	11	10	7	9	7
Outliers	-	-	-	-	-	-	-	-
**Kidney PSR**								
Animals included	9	11	11	10	12	8	9	9
Outliers	-	-	-	-	-	-	-	-

**LA diameter**								
Animals included	9	10	6	8	9	6	7	7
Outliers	1	-	-	-	-	-	-	-
**LVEDD**								
Animals included	9	10	6	8	9	6	7	7
Outliers	-	-	-	-	-	-	-	-
**LVESD**								
Animals included	9	10	6	8	9	6	7	7
Outliers	1	-	-	-	2	-	-	-
**IVS thickness**								
Animals included	9	10	6	8	9	6	7	7
Outliers	1	-	-	-	2	-	-	-
**Ejection fraction**								
Animals included	9	10	6	8	9	6	7	7
Outliers	-	-	-	-	-	1	-	-
**IVRT**								
Animals included	9	10	6	8	9	6	7	7
Outliers	-	-	1	-	-	-	-	-
**Myocyte area**								
Animals included	11	11	10	10	11	7	8	9
Outliers	1	-	3	-	-	-	-	-
**Heart PSR**								
Animals included	11	11	9	8	8	7	8	7
Outliers	-	1	1	-	-	1	1	1
**Collagen I mRNA**								
Animals included	12	11	11	11	12	8	10	9
Outliers	1	-	1	-	-	1	-	2
**α-MHC mRNA**								
Animals included	12	11	11	11	12	8	10	9
Outliers	2	1	-	-	-	-	-	-
**β-MHC mRNA**								
Animals included	12	11	11	11	12	8	10	9
Outliers	-	-	-	1	-	-	-	-
**α-MHC/β-MHC**								
Animals included	12	11	11	11	12	8	10	9
Outliers	1	-	1	-	1	1	1	1
**AGT mRNA**								
Animals included	12	11	11	11	12	8	10	9
Outliers	1	-	-	-	1	-	-	-
**Urine AGT/Creatinine**								
Animals included	5	8	11	10	10	8	10	7
Outliers	1	1	1	1	-	-	2	-
**Serum AGT**								
Animals included	12	10	11	11	12	8	10	9
Outliers	-	-	-	-	-	-	-	-
**Renin mRNA**								
Animals included	12	11	11	11	12	8	10	9
Outliers	-	-	-	-	-	1	-	2
**Renin staining**								
Animals included	9	9	8	9	9	8	5	7
Outliers	-	-	-	1	-	1	-	-
**ACE mRNA**								
Animals included	12	11	11	11	12	8	10	9
Outliers	1	-	1	1	1	-	1	1
**ACE2 mRNA**								
Animals included	12	11	11	11	11	8	10	9
Outliers	1	-	1	-	-	-	2	-
**ACE2/ACE mRNA**								
Animals included	12	11	11	11	11	8	10	9
Outliers	1	1	-	-	-	-	-	-
**ACE act. kidney**								
Animals included	12	11	10	11	12	8	10	9
Outliers	-	1	-	-	-	-	1	-

**ACE2 act. kidney**								
Animals included	12	11	10	11	12	8	10	9
Outliers	-	-	-	-	1	1	-	1
**ACE2/ACE act. ki.**								
Animals included	12	11	10	11	12	8	10	9
Outliers	-	-	-	-	-	-	-	2
**ACE act. serum**								
Animals included	12	11	11	11	11	8	10	8
Outliers	1	-	-	-	2	-	1	-
**ACE2 act. serum**								
Animals included	12	11	11	11	12	8	10	8
Outliers	-	-	-	1	-	1	-	-
**ACE2/ACE act. serum**								
Animals included	12	11	11	11	11	8	10	8
Outliers	-	-	-	-	1	2	-	-

BP: blood pressure. **GFR**: glomerular filtration rate. **UACR**: urine albumin-to-creatinine ratio. **PSR**: picrosirius red staining. **LA**: left atrium. **LVEDD**: left ventricle end-diastolic diameter. **LVESD**: left ventricle end-systolic diameter. **IVS**: interventricular septum. **IVRT**: isovolumetric relaxation time. MHC: myosin heavy chain. **AGT**: angiotensinogen. **ACE**: angiotensin-converting enzyme. **ACE2**: angiotensin-converting enzyme 2.

FIGURES

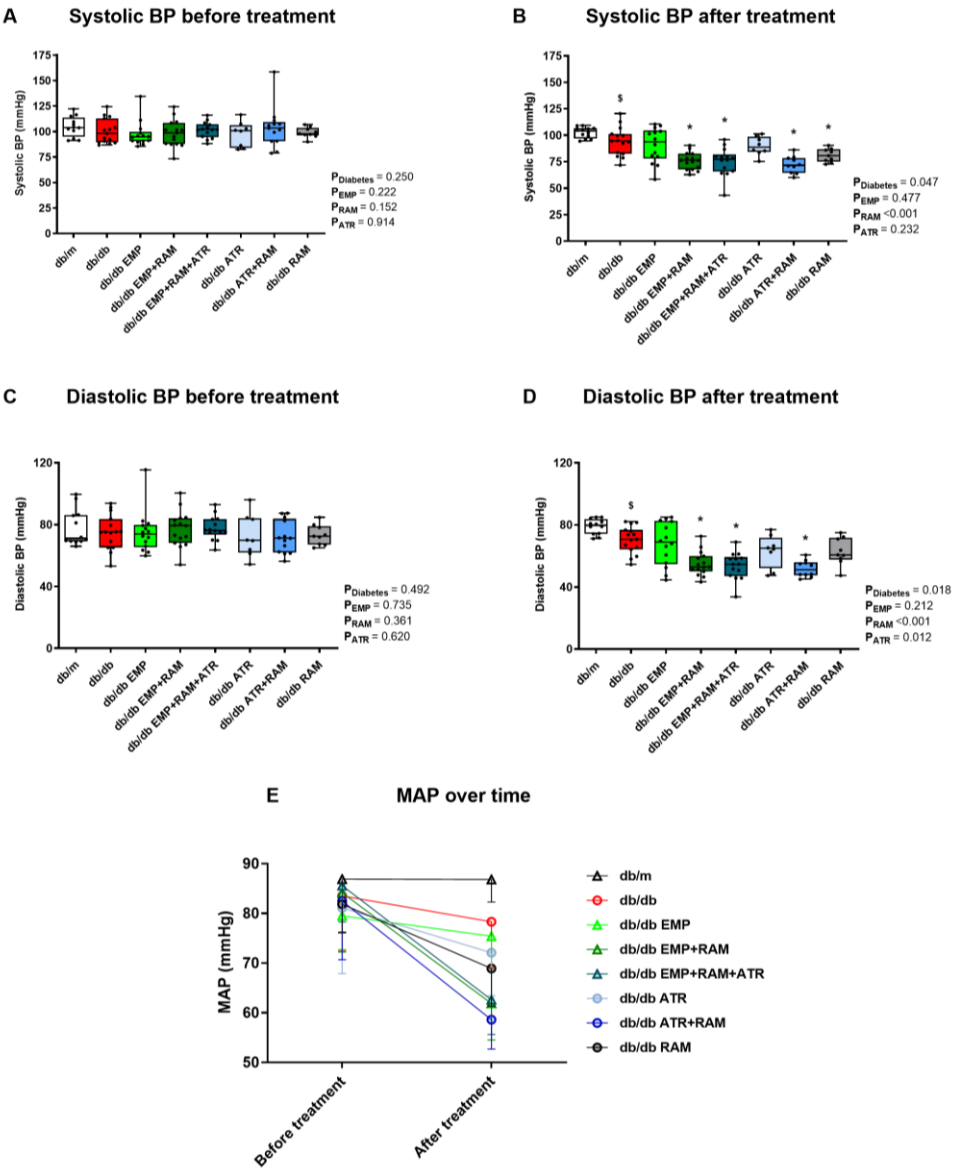

Figure S1. Blood pressure before and after treatment in vehicle-treated db/m mice, vehicle-treated db/db mice and db/db mice treated with empagliflozin, atrasentan, ramipril or their combinations.

Blood pressure was measured in mmHg. (**A**) systolic BP before initiating treatment. (**B**) systolic BP after treatment. (**C**) diastolic BP before initiating treatment. (**D**) diastolic BP after treatment. (**E**) Mean arterial pressure (MAP) before and after treatment. **db/m**: non-diabetic mice treated with vehicle. **db/db**: diabetic mice treated with vehicle. **db/db EMP**: diabetic mice treated with empagliflozin. **db/db EMP+RAM**: diabetic mice treated with empagliflozin and ramipril. **db/db EMP+RAM+ATR**: diabetic mice treated with empagliflozin, ramipril and atrasentan. **db/db ATR**: diabetic mice treated with atrasentan. **db/db ATR+RAM**: diabetic mice treated with atrasentan and ramipril. **db/db RAM**: diabetic mice treated with ramipril. Factorial ANOVA main effect results are displayed next to the graph. **P**$_{\text{Diabetes}}$: diabetes' main effect. **P**$_{\text{EMP}}$: empagliflozin's main effect. **P**$_{\text{RAM}}$: ramipril's main effect. **P**$_{\text{ATR}}$: atrasentan's main effect. $^{\$}p<0.05$ vehicle-treated db/db mice vs vehicle-treated db/m mice. *$p<0.05$ any treated db/db mice compared with vehicle-treated db/db mice.

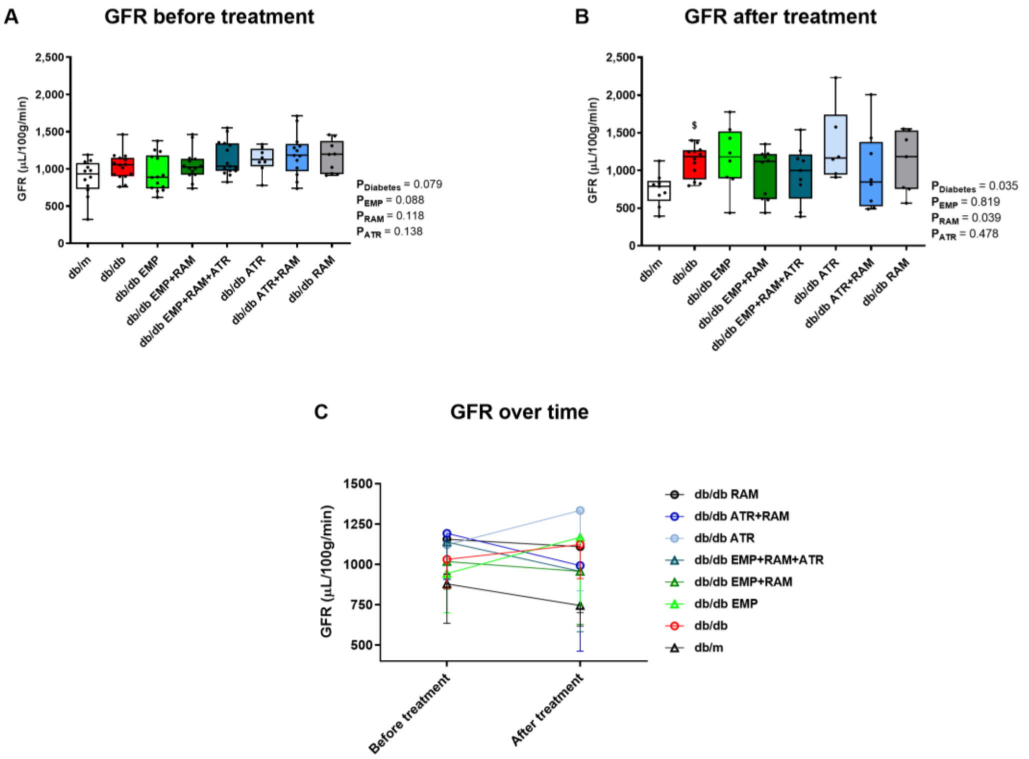

Figure S2. Glomerular filtration rate before and after treatment in vehicle-treated db/m mice, vehicle-treated db/db mice and db/db mice treated with empagliflozin, atrasentan, ramipril or their combinations.

Glomerular filtration rate (GFR) was measured in µL/100g/min. (**A**) GFR before initiating treatment. (**B**) GFR after treatment. (**C**) GFR change and slope after 8 weeks of treatment. **db/m**: non-diabetic mice treated with vehicle. **db/db**: diabetic mice treated with vehicle. **db/db EMP**: diabetic mice treated with empagliflozin. **db/db EMP+RAM**: diabetic mice treated with empagliflozin and ramipril. **db/db EMP+RAM+ATR**: diabetic mice treated with empagliflozin, ramipril and atrasentan. **db/db ATR**: diabetic mice treated with atrasentan. **db/db ATR+RAM**: diabetic mice treated with atrasentan and ramipril. **db/db RAM**: diabetic mice treated with ramipril. Factorial ANOVA main effect results are displayed next to the graph. **$P_{Diabetes}$**: diabetes' main effect. **P_{EMP}**: empagliflozin's main effect. **P_{RAM}**: ramipril's main effect. **P_{ATR}**: atrasentan's main effect. $^{\$}p<0.05$ vehicle-treated db/db mice vs vehicle-treated db/m mice.

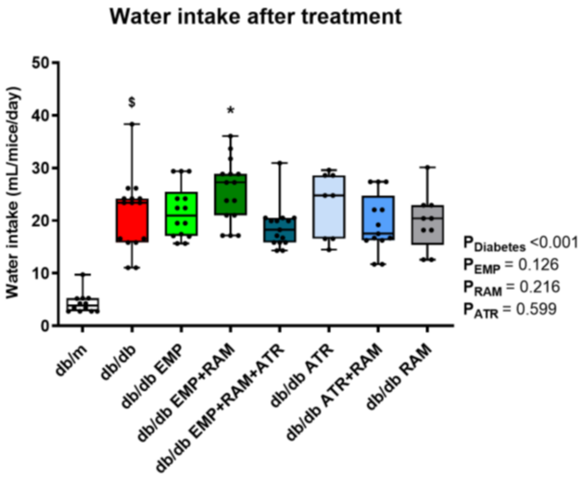

Figure S3. Water intake after treatment in vehicle-treated db/m mice, vehicle-treated db/db mice and db/db mice treated with empagliflozin, atrasentan, ramipril or their combinations. Water intake after treatment measured in mL/mice/day. As expected db/db mice showed increased water intake but it was similar among the seven db/db groups. **db/m**: non-diabetic mice treated with vehicle. **db/db**: diabetic mice treated with vehicle. **db/db EMP**: diabetic mice treated with empagliflozin. **db/db EMP+RAM**: diabetic mice treated with empagliflozin and ramipril. **db/db EMP+RAM+ATR**: diabetic mice treated with empagliflozin, ramipril and atrasentan. **db/db ATR**: diabetic mice treated with atrasentan. **db/db ATR+RAM**: diabetic mice treated with atrasentan and ramipril. **db/db RAM**: diabetic mice treated with ramipril. Factorial ANOVA main effect results are displayed next to the graph. **$P_{Diabetes}$**: diabetes' main effect. **P_{EMP}**: empagliflozin's main effect. **P_{RAM}**: ramipril's main effect. **P_{ATR}**: atrasentan's main effect. $^{\$}p<0.05$ vehicle-treated db/db mice vs vehicle-treated db/m mice. $^{*}p<0.05$ any treated db/db mice compared with vehicle-treated db/db mice.

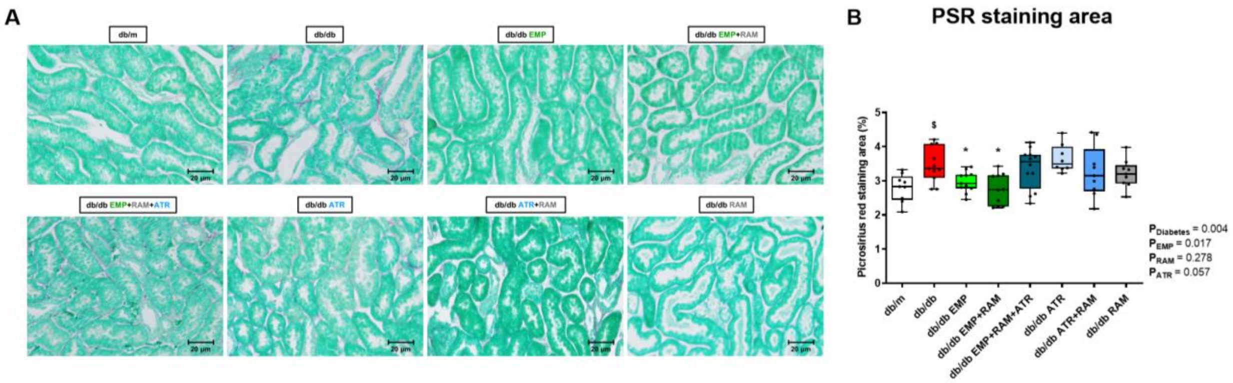

Figure S4. Renal tubulointerstitial fibrosis measured by picrosirius red staining in vehicle-treated db/m mice, vehicle-treated db/db mice and db/db mice treated with empagliflozin, atrasentan, ramipril or their combinations.

(**A**) representative 400x magnification images of renal tubules stained with picrosirius red (PSR). (**B**) Interstitial PSR quantification. **db/m**: non-diabetic mice treated with vehicle. **db/db**: diabetic mice treated with vehicle. **db/db EMP**: diabetic mice treated with empagliflozin. **db/db EMP+RAM**: diabetic mice treated with empagliflozin and ramipril. **db/db EMP+RAM+ATR**: diabetic mice treated with empagliflozin, ramipril and atrasentan. **db/db ATR**: diabetic mice treated with atrasentan. **db/db ATR+RAM**: diabetic mice treated with atrasentan and ramipril. **db/db RAM**: diabetic mice treated with ramipril. Factorial ANOVA main effect results are displayed next to the graph. $\mathbf{P}_{\mathbf{Diabetes}}$: diabetes' main effect. $\mathbf{P}_{\mathbf{EMP}}$: empagliflozin's main effect. $\mathbf{P}_{\mathbf{RAM}}$: ramipril's main effect. $\mathbf{P}_{\mathbf{ATR}}$: atrasentan's main effect. $^{\$}p<0.05$ vehicle-treated db/db mice vs vehicle-treated db/m mice. $^{*}p<0.05$ any treated db/db mice compared with vehicle-treated db/db mice.

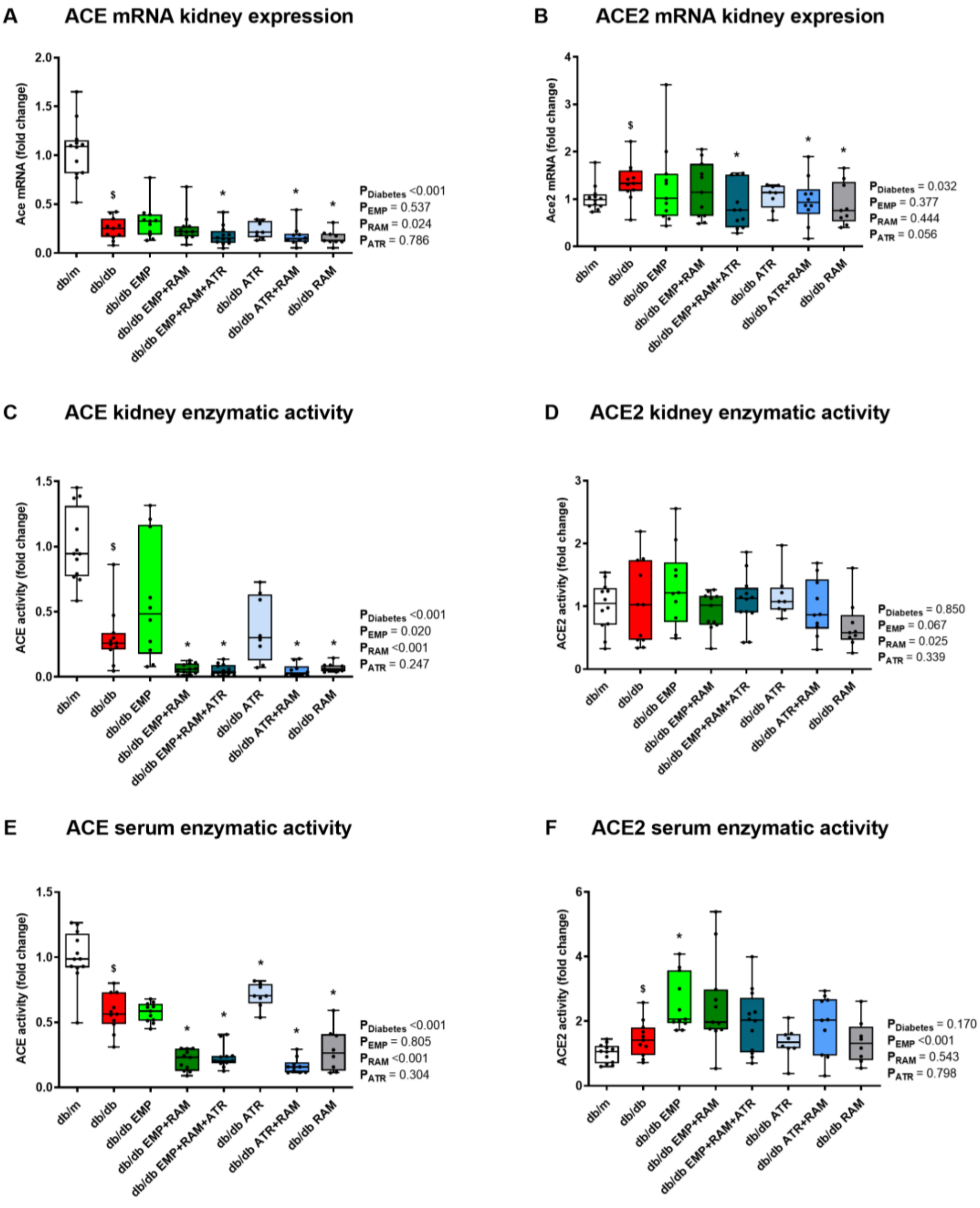

Figure S5. Evaluation of renal mRNA expression and kidney and serum activity of angiotensin-converting enzyme (ACE) and angiotensin-converting enzyme 2 (ACE2) in vehicle-treated db/m mice, vehicle-treated db/db mice and db/db mice treated with empagliflozin, atrasentan, ramipril or their combinations.

(**A**) ACE renal mRNA expression adjusted to db/m mice. (**B**) ACE2 renal mRNA expression adjusted to db/m mice. (**C**) ACE kidney activity adjusted to db/m mice. (**D**) ACE2 kidney activity adjusted to db/m mice. (**E**) ACE serum activity adjusted to db/m mice. (**F**) ACE2 serum activity adjusted to db/m mice. **db/m**: non-diabetic mice treated with vehicle. **db/db**: diabetic mice treated with vehicle. **db/db EMP**: diabetic mice treated with empagliflozin. **db/db EMP+RAM**: diabetic mice treated with empagliflozin and ramipril. **db/db EMP+RAM+ATR**: diabetic mice treated with empagliflozin, ramipril and atrasentan. **db/db ATR**: diabetic mice treated with atrasentan. **db/db ATR+RAM**: diabetic mice treated with atrasentan and ramipril. **db/db RAM**: diabetic mice treated with ramipril. Factorial ANOVA main effect results are displayed next to the graph. $\mathbf{P}_{Diabetes}$: diabetes' main effect. $\mathbf{P}_{EMP}$: empagliflozin's main effect. $\mathbf{P}_{RAM}$: ramipril's main effect. $\mathbf{P}_{ATR}$: atrasentan's main effect. $^{\$}p<0.05$ vehicle-treated db/db mice vs vehicle-treated db/m mice. *p<0.05 any treated db/db mice compared with vehicle-treated db/db mice.

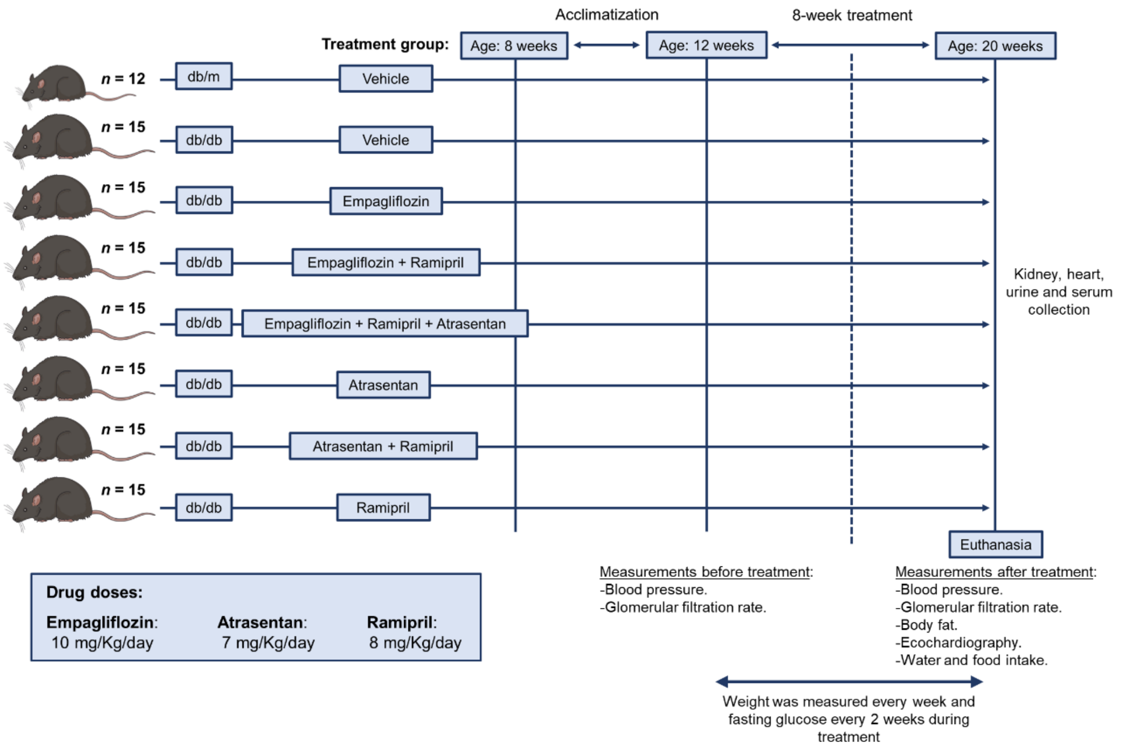

Figure S6. Experimental design.
Scheme of the experimental design of the study which details the time at which treatment was started and ended, as well as the number of mice initially allocated to each of the treatment branches, the measurements performed during the study and the samples collected after mice euthanasia.

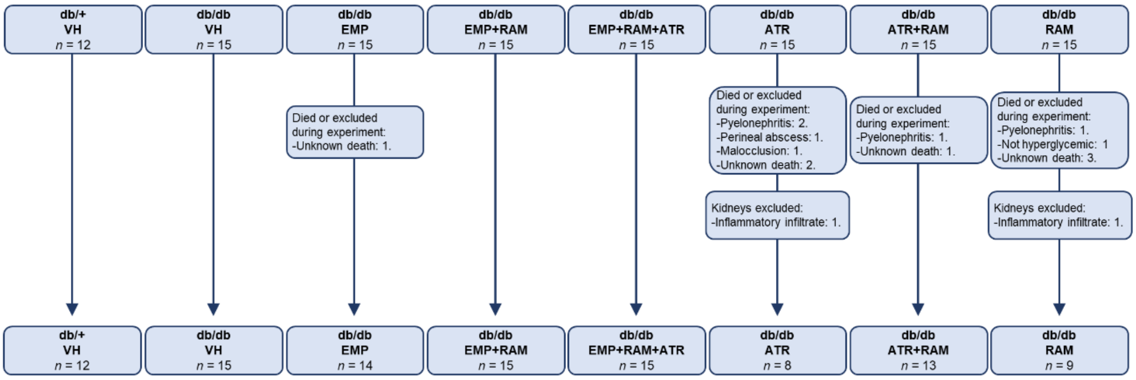

Figure S7. Mice death or excluded during the experimental procedure.
Scheme of the mice included in each group at the beginning of the experiment and the reasons why mice died or were excluded during the conduct of the study.
